# Temporal dynamics of effort discounting: The roles of cognitive load and depression

**DOI:** 10.3758/s13415-026-01410-8

**Published:** 2026-02-14

**Authors:** Yang Yang, Clay Holroyd

**Affiliations:** Department of Experimental Psychology, Ghent University, Henri Dunantlaan 2, 9000 Ghent, Belgium

**Keywords:** Cognitive effort, Effort discounting, Depression, Temporal dynamics, Motivation

## Abstract

Effort discounting, or the devaluation of rewards requiring effort, depends on underlying neural computations of reward valuation and cognitive control. Although much is known about effort discounting, its temporal dynamics—how effort valuation changes when required effort is delayed—remain underexplored. Furthermore, although depression has been linked to altered effort and reward discounting, its role in the temporal dynamics of effort valuation remains unclear. In this study, we conducted three experiments to examine how temporal delays alter effort aversion, with a focus on cognitive load and individual differences in depression. In Experiment 1, we evaluated effort valuation by using the Cognitive Effort Discounting (COG-ED) paradigm together with a backward-typing task and found that effort discounting decreases over time for tasks that require high effort. Experiment 2 replicated this pattern by using a working memory (n-back) task, confirming that effort demand modulates temporal effort discounting for high cognitive loads. Experiment 3 investigated whether depressive traits modulate temporal effort discounting. Nondepressed participants showed strong effects of delay at higher effort levels, with effort discounting decreasing over time. In contrast, depressed participants exhibited reduced sensitivity to delay. Hierarchical drift diffusion modeling (HDDM) revealed that nondepressed individuals flexibly adjusted their decision-making by shifting from positive (high-effort) to negative (low-effort) drift rates as effort increased. In contrast, depressed individuals exhibited consistently negative drift rates across all effort levels, indicating a persistent bias toward effort avoidance. Taken together, these findings highlight how cognitive load influences effort valuation over time and suggest that depression blunts temporal sensitivity in effort-based decisions.

**Supplementary Information:**

The online version contains supplementary material available at 10.3758/s13415-026-01410-8.

## Introduction

Mental effort is often experienced as aversive, as individuals tend to minimize cognitive effort in accordance with the law of least work (Kool & Botvinick, 2018). A recent meta-analysis confirms a robust positive association between mental effort and negative affect across diverse populations and tasks, reinforcing the view that cognitive effort is inherently costly and typically unpleasant (David et al., 2024). Beyond this, cognitive effort has been hypothesized to incur biological costs in which high-control states recruit excessive amounts of metabolic resources that, in turn, result in neural waste accumulation, thereby discouraging prolonged exertion (Holroyd, 2016, 2025). In addition, from a clinical perspective, the cognitive costs associated with mental effort often interfere with persistence in demanding tasks, particularly in neuropsychiatric conditions (Wolpe et al., 2024).

Given these inherent costs of cognitive exertion, individuals often devalue rewards that require effort, which is a phenomenon termed effort discounting (Kool et al., 2010). Westbrook et al. (2013) formalized a procedure for assessing cognitive effort discounting using the Cognitive Effort Discounting paradigm (COG-ED), which used a behavioral economic approach of revealed preferences to quantify subjective effort. In this framework, participants make a series of choices between a low-effort/low-reward option and a high-effort/high-reward option, with effort manipulated through cognitive load (e.g., 1-back vs. 3-back tasks). Their findings demonstrated a clear, parametric relationship: as task difficulty increased, participants exhibited steeper effort discounting (Westbrook et al., 2013). However, effort discounting is not solely determined by task demands. For example, one study found that participants who scored high on the trait Need for Cognition (indicating a greater willingness to engage in cognitive effort activities) perceived more difficult n-back tasks as being less aversive and were more willing to accept higher effort costs compared with those with low need for cognition (Zerna et al., 2023). In addition, previous studies have revealed effort discounting is altered in various clinical populations, with schizophrenia associated with ventral striatum hypoactivation, leading to greater effort cost sensitivity and amotivation (Prettyman et al., 2021). Similarly, depression is characterized by steeper cognitive effort discounting, with reduced willingness to exert effort in some studies (Ang et al., 2023; Westbrook et al., 2023). Given these mixed findings, the role of cognitive effort discounting in depression remains to be fully explained (Barch et al., 2023; Horne et al., 2021).

Although effort discounting has been extensively studied, its temporal dynamics—how willingness to exert effort changes as the time to perform the effortful task approaches—remain relatively unexplored. Theoretical models of optimal decision-making typically assume dynamic consistency, where preferences remain stable over time. However, empirical studies on delay discounting of reward reveal time-inconsistent preferences: individuals discount rewards more steeply as rewards become imminent (Green et al., 1981, 1994; Read et al., 1999). For instance, people often prefer immediate smaller rewards over delayed larger rewards when the reward is imminent but reverse preferences when both options are delayed. Similarly, recent evidence suggests that effort-based decisions also exhibit temporal inconsistency, with people initially planning to undertake effortful tasks but increasingly avoiding them as they become imminent. For example, Augenblick et al. (2015) found that participants allocated more effort to an earlier time when tasks were set further in the future but postponed effort when it was more imminent. In addition, Johnson and Most (2023) found that as an effortful task becomes closer in time, people discount the associated reward more steeply, devaluing rewards when they require immediate effort. These studies suggest that effort-based decision-making involves not only the evaluation of effort-reward trade-offs but also critically depends on exactly when the effort is required.

However, the few, existing studies on temporal effort discounting have some notable methodological limitations. First, in several of these studies the time of effort exertion was confounded with the time of reward delivery, which makes it unclear whether the observed effects were driven by the temporal shift in perceived effort or by the temporal shift in reward anticipation (Augenblick et al., 2015; Johnson & Most, 2023; Le Bouc & Pessiglione, 2022). Second, the rewards were either delivered probabilistically or were entirely hypothetical. As a consequence, only a small subset of participants—or in some of the studies, none of the participants—were actually required to perform the effortful task and then receive the reward for doing so (Le Bouc & Pessiglione, 2022; Vinckier et al., 2022). This task design might have compromised the participants’ belief in the consequences of their choices and therefore their choice valuation. Notably, previous research has shown that individuals tend to value real rewards differently from hypothetical rewards (Horn & Freund, 2022; Scholl et al., 2015). Third, the number of timepoints used to assess delay was typically small. For instance, Johnson and Most (2023) tested only three delays (immediate, 1 day, 1 month) and found no significant difference between the latter two, raising the possibility that discounting effects may unfold over more intermediate temporal scales.

To address these questions, we conducted three experiments that 1) explicitly disentangled effort exertion from reward delivery, 2) ensured that all of the participants experienced real consequences of their choices rather than hypothetical ones, and 3) employed a more fine-grained temporal sampling of delay intervals. Specifically, we included six distinct timepoints: immediate, 1 day, 3 days, 1 week, 2 weeks, and 1 month. These intervals span a realistic short- to medium-term planning range, enabling a more detailed characterization of how subjective effort valuation evolves over time. In Experiment 1, we used an adapted version of the COG-ED paradigm to vary the cognitive demand of a backward-typing task, which had been employed previously in a study on temporal effort discounting (Johnson & Most, 2023). In line with that study, we replaced the typical low-effort baseline (e.g., 1-back) with a no-effort option, thereby anchoring the effort continuum at its minimal point. We adopted the same structure across all three experiments, preserving the core titration logic of the paradigm while enabling estimation of subjective value across the full range of effortful task conditions—including task levels (e.g., 1-back) that traditionally functioned as the low-effort baseline. This allowed us to examine not only how much effort participants were willing to exert but also whether they were willing to engage in any cognitive effort at all—a distinction particularly relevant for our clinical comparison in Experiment 3. Our goal in Experiment 1 was to replicate and extend those findings following the methodological changes described above. Notably, although Experiment 1 allowed us to assess whether temporal cognitive effort discounting occurs, the backward-typing task conflates increases in cognitive demand with increases in both physical exertion and task duration, which can influence participants’ choices independently of effort discounting. Furthermore, given that higher cognitive load has been shown to intensify effort discounting (Westbrook et al., 2013), we hypothesized that temporal inconsistency—the tendency to discount immediate effort more steeply than delayed effort—might only emerge, or become more pronounced, under higher cognitive load. To better isolate the effect of cognitive demand, Experiment 2 utilized the n-back task, which is a well-established, more cognitively demanding task that controls for physical effort and task duration across effort levels (Westbrook et al., 2013).

Finally, despite considerable research on the relationshi*p* between effort discounting and various mental disorders (Barch et al., 2023; Chong et al., 2016), no prior study has specifically examined the temporal dynamics of cognitive effort discounting in individuals with depression. Depression has been linked to both heightened effort aversion and greater future reward discounting (Amlung et al., 2019; Westbrook et al., 2023), but these findings are mixed. One potential explanation for the discrepancy is that motivational impairments in depression may differentially affect the valuation of immediate vs. delayed effort due to impairments in goal initiation (Grahek et al., 2019) or deficits in episodic future thinking (Hallford et al., 2020). These mechanisms could contribute to temporal inconsistencies in how effort costs are evaluated. In Experiment 3, we therefore examined whether this temporal dimension of effort discounting could provide insight into the motivational deficits associated with depression. To address this, we compared individuals with severe depressive symptoms to those with minimal symptoms, as defined by established clinical cutoffs. This categorical design allowed us to maximize statistical sensitivity for detecting group-level effects, providing an initial test of whether depression alters how effort costs unfold over time. Furthermore, to examine whether the two groups differed in their internal evidence accumulation processes during effort-based decision-making, we applied hierarchical drift diffusion modeling (HDDM) to the behavioral data. By clarifying the distinctions between motivational impairments in depression and those in other psychiatric disorders, these results could facilitate the development of targeted diagnostic tools and personalized interventions used in precision psychiatry.

## Experiment 1

As noted in the introduction, the present study further developed the methodological procedures used in a previous study on temporal discounting of cognitive effort. In particular, building on the work of Johnson and Most (2023), we implemented design changes that 1) disentangled experimental effects related to the relative timing of effort exertion vs. reward delivery, 2) provided more granular observations of temporal delay, and 3) ensured that each participant received a real monetary reward based on their task choices about future effort expenditure.

### Participants

Based on prior research on temporal effort discounting (Johnson & Most, 2023), an effect size of 0.20 was used for a priori power analysis conducted in G*Power 3.1.9.7 (Faul et al., 2007). Assuming α = 0.05, power (1 − β) = 0.80, and 24 repeated measurements per participant (4 effort levels × 6 temporal delays), the analysis indicated a minimum sample size of 13 participants. Because the planned analyses employed linear mixed models (LMMs), which are less sample-size dependent for detecting fixed effects and can efficiently handle missing or unbalanced data (Muth et al., 2016; Schad et al., 2020), this sample size remains valid. To accommodate potential data exclusions or attrition, and to enhance the precision of fixed effect estimates, we recruited 40 participants (mean age = 30.5 years, standard deviation [SD] = 6.68; 19 males) via Prolific, an online crowdsourcing platform commonly used for academic research. Participants were required to be fluent in English and older than 18 years. The research was conducted in accordance with the General Ethics Protocol of the Faculty of Psychology and Educational Sciences of Ghent University, and all participants provided informed consent prior to participation.

### Design and procedure

Experiment 1 employed a within-subject design with four effort levels (50, 100, 150, 200 words) and six temporal delays (immediate, 1 day, 3 days, 1 week, 2 weeks, 1 month). Effort was manipulated by using a backward typing task, where participants typed a specified number of words in reverse order. To separate the timing of effort exertion from reward delivery, participants were informed that all monetary rewards—regardless of when the effort was scheduled—would be delivered exactly 1 month (the maximum effort delay) after the experiment. This approach ensured that the participants would fully engage with the effortful choices, free from any confounds due to differences in reward delivery timing.

At the beginning, participants were informed that one of their choices from the upcoming task would be randomly selected and that they would be required to complete the chosen effort at the specified time in order to receive the corresponding reward. To allow for scheduling flexibility, the selected task could be completed at any point within a 2-day window starting from the scheduled date. This instruction aimed to ensure that participants perceived their decisions as being meaningful as regards the stated incentives. Following this instruction, participants completed two self-report measures: the Need for Cognition Scale (NCS; Cacioppo & Petty, 1982) and the Pure Procrastination Scale (PPS; Rebetez et al., 2014). The NCS is an 18-item measure that quantifies an individual’s intrinsic motivation to engage in cognitively demanding tasks, with scores ranging from − 72 to + 72, where higher values indicate greater enjoyment in cognitively effortful activities. The PPS is a 12-item scale that assesses the tendency to voluntarily postpone tasks and experience difficulties meeting deadlines, with responses recorded on a 5-point Likert scale, and higher scores reflecting greater procrastination tendencies.

**Fig. 1:**
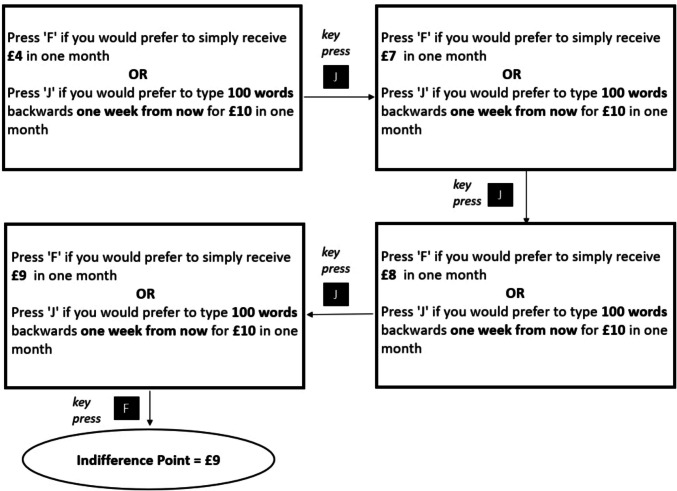
Hypothetical example of finding the SV of typing 100 words 1 week from now for £10. Indifference point is the index of SV. The value of the smaller reward requiring no effort was systematically adjusted via a staircase procedure based on the participant’s responses. In this example, the staircase algorithm stopped, because 1) the participant preferred the larger, effortful reward when the smaller, effortless reward was £8, and 2) when the smaller, effortless reward was just £1 different (£9), the participants preference changed to the smaller, effortless reward. Therefore, the participant is said to be indifferent between the rewards when the smaller, effortless reward is £9

Following the questionnaire administration, participants were asked to complete a practice round consisting of a 50-word backward typing task to familiarize themselves with the task demands. Afterward, they proceeded to the effort discounting task, which involved a series of binary choices between a smaller monetary reward requiring no effort and a larger monetary reward that required effort (fixed at £10), with both effort level and effort timing varying across trials. This reward amount was selected following pilot testing: although we initially adopted the £20 reward used in Johnson and Most (2023), this value led to ceiling effects in choice behavior—possibly due to the more credible reward implementation in our design, where every participant (rather than a randomly chosen few) received an actual payout. To maintain decision sensitivity, we reduced the reward to £10 and expanded the effort range beyond the original levels used in Johnson and Most (2023), as detailed above. An example trial presented the options: “Simply receive £5 in 1 month” vs. “Perform a 150-word backward typing task tomorrow for £10 in 1 month.” The amount of the smaller reward was dynamically adjusted using a staircase algorithm to identify each participant’s indifference point—the value at which they had no clear preference between the easy and hard options. The procedure stopped when a reversal in preference occurred across a £1 difference (Fig. 1). From this, subjective value (SV) was computed as the indifference point divided by £10 (which is the amount of the reward associated with task); hence, higher SV indicated lower effort discounting.

To control for order effects, the sequence of effort levels and temporal delays was randomized for each participant. At the conclusion of the experiment, participants were informed which of their choices had been randomly selected. Rather than requiring them to complete the corresponding task at the date specified by that choice, they were instead compensated with the monetary value corresponding to their indifference point—that is, the amount they were willing to accept to avoid exerting the effort. Participants were notified that this payment would be credited to their Prolific account exactly 1 month later.

At the end of the experiment, participants answered several brief questions assessing their subjective experience of the backward typing task (e.g., “How fun did you find the 200-word backward typing task?” and “How effortful did you find the 200-word backward typing task?”).

### Statistical analysis

Across all three experiments, effort discounting was analyzed by using linear mixed-effects models (LMMs) to account for both fixed effects of experimental manipulations and random effects due to individual variability. All analyses were conducted in R (v.4.3.2), with SV as the dependent variable.

Experimental factors were coded using treatment contrasts, allowing each level to be compared against a designated reference level. Reference levels were selected based on our hypothesis that temporal effort discounting—the increase in SV with delay—would primarily emerge at higher effort levels. To test this hypothesis directly, we set the highest effort level and the immediate ("right now") timepoint as reference levels (Schad et al., 2020). This setup facilitated a preliminary evaluation of the hypothesized temporal effect under maximally demanding conditions, where it was expected to manifest most clearly. For the design of the random effect structure, we initially specified a maximal random-effects structure, including by-subject random intercepts and slopes. To ensure model convergence and prevent overparameterization, random-slope correlations were set to zero when necessary, and slopes explaining zero variance were omitted (Bates et al., 2015). The significance of fixed effects was assessed by using the Satterthwaite approximation via the lmerTest package (Kuznetsova et al., 2017); *p* <.05 was considered statistically significant.

Given the complex factorial design (e.g., 6 effort levels × 6 time delays in Experiment 2), a full factorial interaction yields a large number of comparisons, many of which are not theoretically meaningful (e.g., SV at effort level 2 in 1 day vs. effort level 6 right now). Following Schad et al. (2020), we adopted a hypothesis-driven contrast strategy that focused on planned pairwise comparisons across time delays within each effort level, rather than interpreting global interaction terms in the LMM. This approach allows for statistically efficient and theory-consistent testing without requiring a significant overall interaction as a precondition for targeted comparisons. Thus, after the initial LMM analysis, we conducted planned pairwise comparisons (Tukey-adjusted) to examine temporal delay effects within each effort level, directly assessing our prediction regarding effort-dependent temporal discounting.

Moreover, to explore individual differences in the temporal effort discounting context, we conducted Spearman correlation analysis between questionnaire variables (e.g., NCS) and SV.

### Results

#### Linear mixed model analysis

**Fig. 2:**
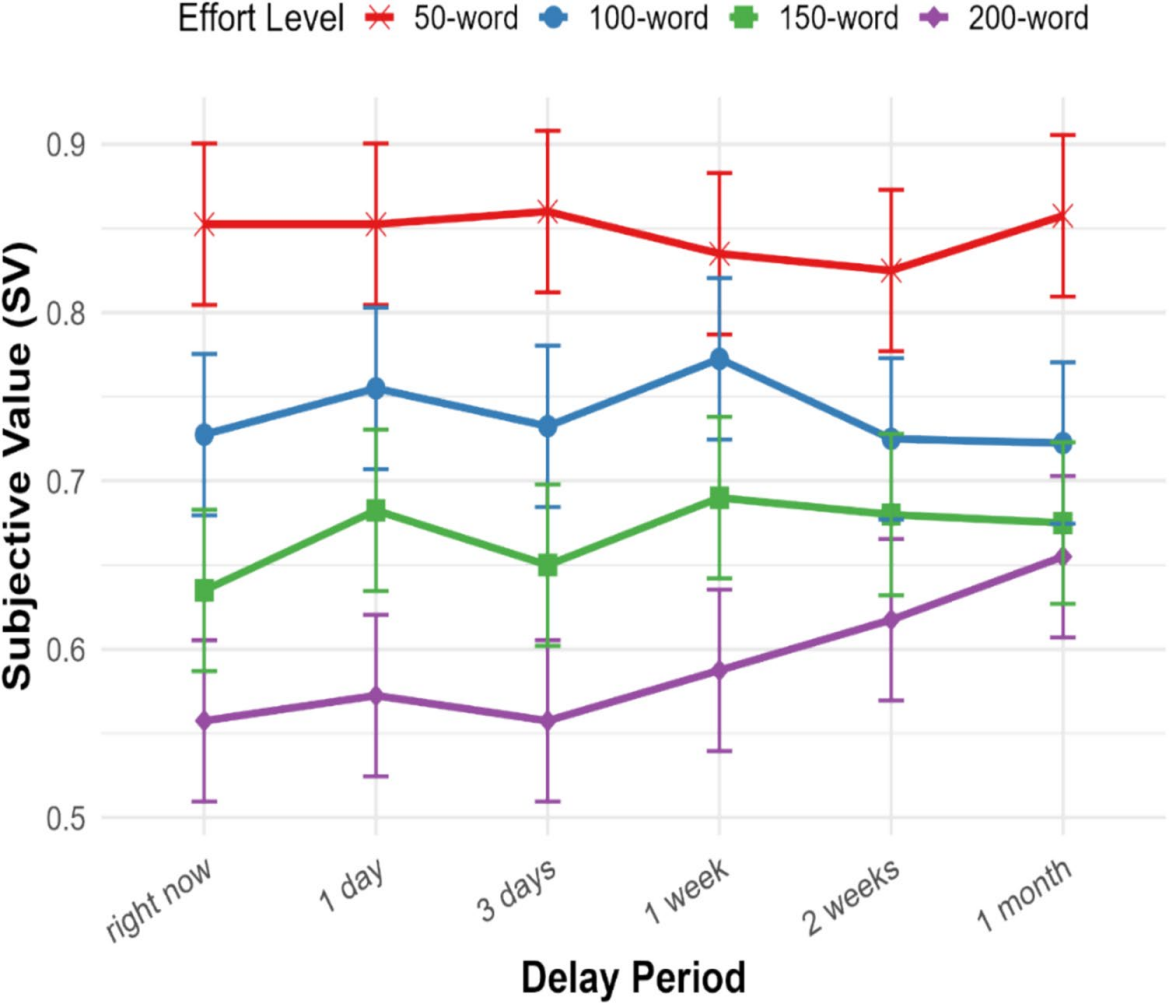
Mean SV across temporal delays for each effort level in Experiment 1. Changes in SV (y-axis) as a function of the temporal distance to effort exertion (X-axis: from “right now” to “1 month”) across four effort levels (50-word to 200-word represents an increasing cognitive load, color-coded). Error bars represent ± 1 standard error of the mean

The final linear mixed model included effort level, effort timing, and their interaction as fixed effects, with random intercepts for subjects as a random effect. According to the fixed-effect results, there was a time delay effect under the highest effort level (200-word): a significant difference in SV was observed at the "1 month” delay compared with “right now” (*p* =.004). Further pairwise comparisons revealed that no significant differences were found for lower effort conditions (50-word to 150-word; *p*s >.05) (Fig. 2). However, for the highest effort condition (200-word), SV increased significantly from timepoint “right now” to timepoint “in 1 month” (*p* =.045) as well as from timepoint “in 3 days” to timepoint “in 1 month” (*p* =.045). All other comparisons within the highest effort level were not significant. These findings suggest that while SV remains stable over time for lower effort tasks, high cognitive effort is discounted less when scheduled further in the future. The detailed output of the linear mixed-effects model is available in the supplementary materials (Table S1).

#### Individual differences in effort valuation

Furthermore, to explore individual differences in the context of temporal effort discounting, we conducted Spearman correlation analyses between mean SV and several individual variables, including NCS, PPS, perceived effort, and perceived fun for both low- (50-word) and high-effort (200-word) tasks. A key finding was that fun ratings for both the low-effort and high-effort tasks were significantly positively correlated with mean SV (low-effort task: r = 0.39, *p* =.010; high-effort task: r = 0.43, *p* =.006), suggesting that participants who found the tasks more enjoyable also tended to assign greater SV to effort-based rewards. This result highlights the potential influence of intrinsic motivation on cognitive effort valuation: individuals who found the tasks to be engaging were also more willing to expend effort for rewards. No significant correlations were found between mean SV and the other variables (NCS, PPS, or perceived effort ratings), indicating that task enjoyment, rather than perceived effort or trait measures, was the primary factor associated with individual differences in effort valuation.

### Discussion

Experiment 1 replicated prior findings that increasing effort reduces SV, consistent with robust cognitive effort discounting. Crucially, temporal delay increased SV only at the highest effort level, suggesting that future cognitive effort is discounted less steeply, but only when the anticipated demand is substantial. One possible explanation for the selective delay effect is that under low-effort conditions, participants were less willing to sacrifice tangible monetary rewards simply to postpone relatively easy tasks.

These findings build on previous work (Johnson & Most, 2023) by providing more granular evidence for an effort-dependent temporal discounting effect. Moreover, our use of real monetary rewards addresses a key methodological question in prior studies that relied on hypothetical scenarios. Finally, the positive correlation between task enjoyment and SV highlights the role of intrinsic motivation in shaping effort-based choices.

## Experiment 2

Importantly, the backward-typing task used in Experiment 1 to manipulate cognitive effort is characterized by relatively low cognitive demand and also confounds cognitive effort with both physical effort and task duration. To address these considerations, in Experiment 2, we employed the n-back task, which yields a well-validated measure of cognitive effort that allows for parametric variation in cognitive load, while also controlling for physical effort and time-on-task (Westbrook et al., 2013).

### Participants

An a priori power analysis as in Experiment 1 based on 36 repeated measures per participant (6 effort levels × 6 timepoints), indicated that ten participants would be sufficient to detect medium effects. To ensure robustness and allow for potential exclusions, 45 participants (mean age = 31.13 years, SD = 6.29; 29 males) were recruited via Prolific. Eligibility criteria were identical to Experiment 1. The research was conducted in accordance with the General Ethics Protocol of the Faculty of Psychology and Educational Sciences of Ghent University, and all participants provided informed consent prior to participation.

### Design and procedure

Experiment 2 employed the same effort discounting paradigm as Experiment 1, with a key modification: effort was manipulated using the n-back working memory task instead of backward typing. The within-subject design included 6 effort levels ranging from 1-back (effort level 1) to 6-back (effort level 6) and six temporal delays (immediate, 1 day, 3 days, 1 week, 2 weeks, 1 month). The 6-back level was included to represent the highest cognitive load, consistent with Westbrook et al. (2013), who used a similar n-back range to manipulate effort in an effort-based decision-making context. As in Experiment 1, the reward delivery was fixed at 1 month postexperiment to isolate the effects of effort timing.

**Fig. 3:**
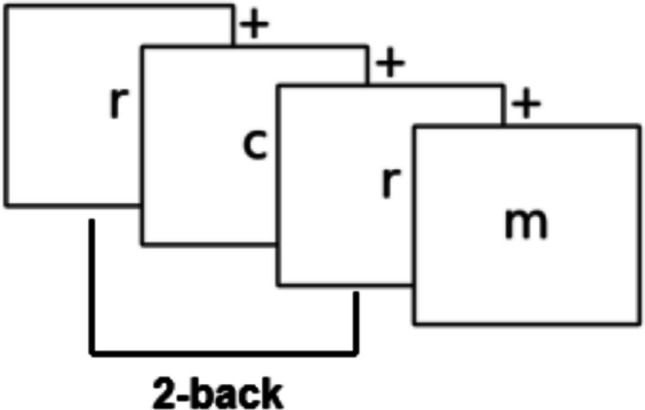
Example trial sequence in a 2-back task. Participants view a continuous stream of letters and indicate whether the current letter matches the one presented two trials earlier. In this example, the letter "r" is a 2-back match. A fixation cross (" + ") is displayed between stimuli to maintain attention

In the n-back task, participants viewed a sequence of letters and responded when the current letter matched the one n positions earlier (Fig. 3). Higher n-back levels required greater working memory load, with 1-back corresponding to the lowest and 6-back the highest cognitive demand. During a practice phase, participants completed two practice runs at each level (64 trials per run; 1.5-s response window; 3.5-s interstimulus interval), followed by NASA-TLX ratings assessing subjective task experience (Hart & Staveland, 1988).

The choice phase mirrored Experiment 1, with participants making binary decisions between a smaller, effortless reward and a larger, effortful reward. The larger reward was fixed at £2, consistent with the original Cog-ED paradigm developed by Westbrook et al. (2013), which used this amount and reliably revealed differences in subjective valuation across effort levels.

In each trial, participants were presented with a choice, such as: “Simply receive £1 in 1 month” vs. “Perform a 2-back task tomorrow for £2 in 1 month.” As in the original paradigm, participants were informed that if an effortful option was selected for implementation, they would be required to perform the chosen task three times on the specified future date in exchange for three times the reward amount (e.g., £6). The amount of the smaller reward was adjusted across trials using an adaptive titration procedure modeled after Westbrook et al. (2013). Specifically, if the participant chose the high-effort option, the zero-effort offer increased on the next trial; if they chose the zero-effort option, the offer decreased. After each choice, the adjustment magnitude was halved, producing finer-grained estimates with each successive trial. This procedure was repeated for six trials per condition, and the final offer amount on the sixth trial was taken as the participant’s indifference point.

Subjective value was calculated by dividing the indifference point by the fixed high-effort reward (£2), with lower SVs indicating greater discounting of effort. This six-step titration procedure—despite the small number of trials per condition—has been shown to yield precise subjective valuation estimates with minimal trial burden (Westbrook et al., 2013). The order of the effort levels and the timepoints was randomized across subjects. As in Experiment 1, participants were not required to complete the task post-experiment and were paid 1 month later the amount equal to the indifference point of the selected choice.

### Statistical analysis and result

#### Analysis of task performance and subjective task experience

We first tested for load effects on performance and self-reported indicators as a manipulation check for effort (see the descriptive statistics in Table 1). Specifically, we used linear mixed models to assess the effects of n-back load on task performance (signal detection sensitivity d’ and reaction time RT), as well as all items in NASA-TLX, treating effort level as a continuous numeric predictor and including random intercepts for subjects to account for individual differences.

**Table 1:** Mean (SD) of performance and subjective experience ratings across effort levels in Experiment 2

Effort level	D'	RT (ms)	Mental demand	Perceived effort	Estimated performance	Fun	Frustration	Temporal demand	Physical demand
1	3.70 (1.14)	587.92 (137.15)	7.47 (5.94)	8.96 (5.76)	17.40 (2.51)	9.64 (5.55)	3.62 (4.69)	6.02 (5.28)	3.87 (4.87)
2	2.63 (1.19)	713.34 (144.01)	12.78 (4.79)	13.87 (4.07)	14.93 (3.88)	8.96 (5.56)	5.82 (4.77)	7.73 (5.61)	6.09 (5.96)
3	1.85 (1.16)	801.13 (151.29)	15.07 (3.66)	14.13 (4.10)	12.18 (5.28)	8.80 (5.35)	8.98 (6.21)	8.67 (5.76)	7.36 (6.23)
4	1.38 (1.02)	818.92 (193.96)	16.42 (2.75)	15.11 (3.84)	10.80 (5.21)	7.49 (5.28)	9.67 (5.96)	8.82 (5.87)	7.56 (6.45)
5	1.04 (1.01)	817.91 (192.49)	17.29 (2.13)	16.44 (2.44)	9.89 (5.13)	6.67 (5.57)	11.22 (6.26)	8.91 (6.45)	8.36 (6.79)
6	0.86 (0.89)	849.16 (177.98)	17.71 (1.96)	16.53 (3.06)	8.20 (4.65)	6.04 (4.89)	10.96 (6.29)	9.18 (6.35)	8.02 (6.86)

Effort level 1–6 represents 1-back to 6-back task. Performance metrics include d′ (sensitivity index) and mean response time (RT, ms). Subjective experiences were assessed after each n-back level using the NASA Task Load Index Scale (NASA-TLX), covering mental demand, perceived effort, estimated performance, fun, frustration, temporal demand, and physical demand. Linear mixed model analyses revealed significant linear effects of effort level on all measures (*p* <.001): performance (d′ and RT) worsened with increasing load, while subjective demands increased, and perceived performance and fun decreased

Consistent with prior work (Westbrook et al., 2013), a linear mixed model revealed a significant negative effect of effort level on d' (b =  − 0.562, SE = 0.025, t(249) =  − 22.40, *p* <.001). This observation indicates that as effort levels increased, the participants' ability to discriminate between the target and nontarget stimuli significantly declined. Similarly, a linear mixed model on mean RT showed a significant positive effect of effort level (b = 46.41, SE = 4.39, t(249) = 10.57, *p* <.001), meaning that participants responded more slowly as task difficulty increased.

The results of subjective feelings under each n-back task revealed significant linear effects of effort level on all NASA-TLX dimensions. As expected, higher effort levels were associated with greater subjective task demands. Specifically, effort level had a strong positive effect on perceived mental demand (b = 1.85, SE = 0.11, t(249) = 16.44, *p* <.001), effort required (b = 1.29, SE = 0.11, t(249) = 11.56, *p* <.001), temporal demand (b = 0.54, SE = 0.10, t(249) = 5.60, *p* <.001), frustration (b = 1.52, SE = 0.14, t(249) = 11.10, *p* <.001), and physical demand (b = 0.73, SE = 0.10, t(249) = 7.12, *p* <.001). Additionally, effort levels negatively influenced task performance ratings (b =  − 1.82, SE = 0.12, t(249) =  − 15.25, *p* <.001) and perceived fun (b =  − 0.75, SE = 0.10, t(249) =  − 7.23, *p* <.001), suggesting that participants found higher-effort tasks less enjoyable and perceived their performance as worse as task difficulty increased.

Together, these findings validate the effectiveness of the effort level manipulation and demonstrate the impact of increasing cognitive load on both subjective effort perception and objective performance measures.

#### Linear mixed model analysis

Consistent with the analysis strategy in Experiment 1, a linear mixed-effects model was conducted to examine the effects of effort level and temporal delay on SV. The final model included effort level, effort timing, and their interaction as fixed effects. Random intercepts and random slopes for effort level by subject were included as random effects. Following previous research (Westbrook et al., 2013, 2023), participants’ n-back task performance (d′) was included as a covariate to control for individual differences in task ability.

According to the fixed effect results, under the highest effort level (level 6), we found that SV was significantly affected by the time to effort exertion. Specifically, compared with the present moment, SV significantly increased at later timepoints: in 1 week (b = 0.090, *p* < 0.001), in 2 weeks (b = 0.077, *p* = 0.002), and in 1 month (b = 0.056, *p* = 0.026). Detailed model output can be found in the supplementary materials (Table S2).

**Fig. 4:**
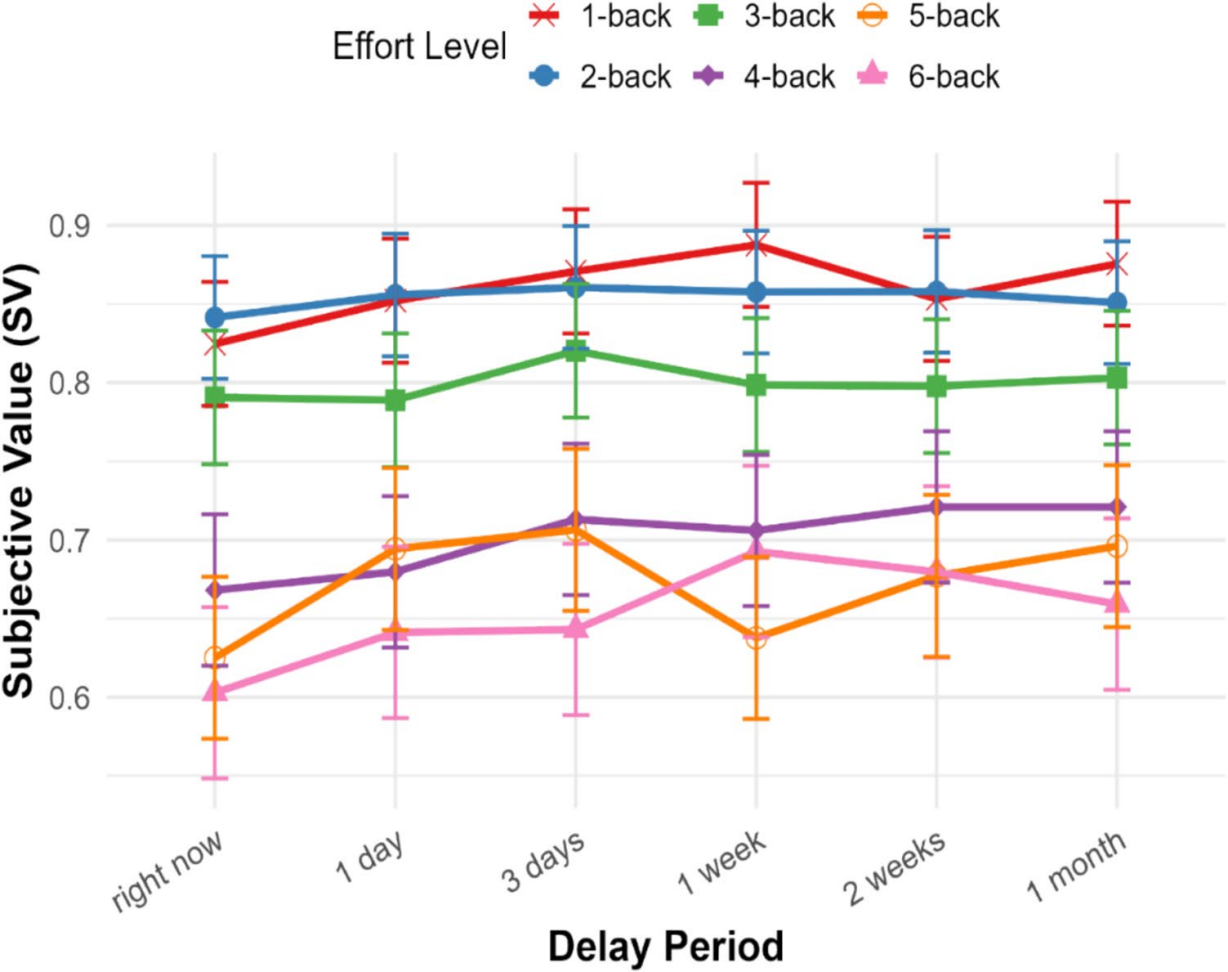
Mean SV across temporal delays for each effort level in Experiment 2. The figure depicts changes in SV as a function of the temporal delay of effort exertion (x-axis: from right now to 1 month) across six effort levels (1-back to 6-back represents an increasing cognitive load, color-coded). Error bars represent ± 1 standard error of the mean

To further explore how SV changes across different time delays under each effort level, we conducted pairwise comparisons. Consistent with Experiment 1, results revealed there were no significant differences among temporal delays under relatively lower effort levels (from effort level 1 to effort level 4) (Fig. 4). For effort level, the SV of doing the 5-back task right now was significantly lower compared with delaying the task by 3 days (b =  − 0.081, *p* = 0.016). Other pairwise comparisons under effort level 5, such as “right now” vs. “in 1 day” (b =  − 0.069, *p* = 0.067) and “right now” vs.”in 1 month” (b =  − 0.071, *p* = 0.056), showed marginal significance. For effort level 6, SV was significantly lower at the present moment compared with delaying the task by 1 week (b =  − 0.089, *p* =.005) and 2 weeks (b =  − 0.077, *p* =.029). Crucially, these results indicate that the effect of temporal delay on effort valuation is effort-dependent, particularly at higher effort levels. Although not all future timepoints showed significantly higher SV compared with the present moment, the general trend suggests that individuals assign greater SV to future effort than present effort, especially at higher effort levels.

#### Individual differences in effort valuation

Additionally, fun ratings for the n-back task were significantly and positively associated with SV (b = 0.042, *p* = 0.004), indicating that participants who perceived the task as more enjoyable also assigned it greater SV. This finding is consistent with Experiment 1, in which fun also positively predicted SV. Also aligning with the results of Experiment 1, individual traits of Need for Cognition and procrastination were not significantly associated with SV, suggesting that these traits do not significantly modulate the effort-reward trade-off, at least in the context of these specific tasks.

### Discussion

In summary, Experiment 2 replicated and extended the findings of Experiment 1 using the n-back task, which allowed for precise manipulation of cognitive load while controlling for physical effort and time-on-task. Importantly, delaying the date of the effortful task increased the SV of the associated reward only for the higher effort levels, which indicates that temporal effort discounting effect is robustly moderated by cognitive load. Moreover, consistent with Experiment 1, task enjoyment is associated with higher SV, highlighting the role of intrinsic motivation underlying individual differences in the effort-reward trade-off.

## Experiment 3

Despite extensive research on effort discounting in various mental disorders, the temporal dynamics of cognitive effort valuation in depression remain unexplored. Moreover, previous findings about the relations between depression and both effort aversion and future reward discounting have been mixed. Here, we focused specifically on temporal effort discounting to gain insight into the motivational impairments underlying depression. As an initial step, we employed a group-based design that contrasted individuals with high versus low-to-minimal depressive symptoms using established cutoff criteria. This categorical approach enabled us to examine clear between-group differences. In particular, Experiment 3 examined whether depressive traits influence the temporal inconsistency in effort valuation identified in Experiments 1 and 2, thereby providing preliminary evidence about how depression affects decisions to delay the execution of effortful tasks.

### Participants

We implemented a screening procedure to identify depressed individuals and nondepressed individuals. Specifically, a total of 600 participants were recruited via Prolific initially. Participants completed two validated self-report measures of depression: the Patient Health Questionnaire-9 (PHQ-9; Kroenke et al., 2001) and the Center for Epidemiologic Studies Depression Scale (CES-D; Radloff, 1977). Based on established clinical cutoffs, individuals scoring ≥ 16 on the CES-D (indicative of depression) and ≥ 15 on the PHQ-9 (indicative of moderate to severe depression) were assigned to the depression group, while those scoring < 16 on the CES-D and ≤ 4 on the PHQ-9 (indicative of no depression) were assigned to the nondepression group. Except for these differences, the eligibility criteria were identical to Experiment 1. Following the questionnaire screening, participants who failed to complete the n-back task practice were excluded. As a result, 50 participants qualified for the depression group, and 58 participants qualified for the nondepression group (see Table 2 for participant characteristics). Additional correlation analysis across these questionnaire variables can be found in the supplementary materials (Fig. S1). The research was approved by the Ethics Committee of the Faculty of Psychology and Educational Sciences of Ghent University. All participants provided informed consent prior to participation.

**Table 2:** Participant characteristics

Variable	Nondepression group (N = 58)	Depression group (N = 50)	*p*
Age [mean (SD)]	36.8 (10.4)	33.2 (10.3)	0.062
Gender (M:F)	33:25	29:21	1.000
PHQ-9 total [mean (SD)]	1.3 (1.4)	18.9 (3.6)	0.000
CES-D total [mean (SD)]	5.3 (3.9)	40.4 (7.5)	0.000
SHAPS total [mean (SD)]	0.9 (2.3)	4.7 (4.4)	0.000
GAD-7 total [mean (SD)]	1.8 (2.8)	15.3 (3.8)	0.000
PPS [mean (SD)]	− 19.8 (22.2)	15.6 (21.1)	0.000
NCS [mean (SD)]	17.1 (21.8)	2.7 (22.4)	0.000

PHQ-9 = Patient Health Questionnaire-9 item; CES-D = Center for Epidemiologic Studies Depression Scale; SHAPS = Snaith Hamilton Pleasure Scale; GAD-7 = Generalized Anxiety Disorder-7; PPS = Pure Procrastination Scale; NCS = Need for Cognition Scale

### Design and procedure

Experiment 3 used the same effort discounting paradigm as Experiment 2, employing a 6 (Effort Level: 1–6-back) × 6 (Temporal Delay: immediate, 1 day, 3 days, 1 week, 2 weeks, 1 month) × 2 (Group: depression, nondepression) mixed design. Effort level and temporal delay were within-subjects factors; group was a between-subjects factor. Monetary rewards were fixed for delivery 1 month after the experiment. In addition to the NCS, PPS, and NASA-TLX used previously, participants completed measures of anhedonia (Snaith-Hamilton Pleasure Scale, SHAPS; Snaith et al., 1995) and anxiety (GAD-7; Spitzer et al., 2006).

Participants were prescreened via PHQ-9 and CES-D questionnaires. Those meeting criteria for the depressed or nondepression group were invited to the main study. The following procedure was identical to Experiment 2, with the addition of SHAPS and GAD-7 questionnaires.

### Computational modeling of decision processes (HDDM)

In addition to behavioral analyses, we applied HDDM to estimate and compare latent decision-making processes in individuals with and without depression (Pan et al., 2025; Wiecki et al., 2013). We initially specified a fully saturated model in which all parameters (drift rate v, boundary separation a, starting point z, nondecision time t) were allowed to vary as a function of group (depression vs. nondepression), effort level (1-back to 6-back) and time delays (“immediate” to “1 month”). All models were estimated using Markov Chain Monte Carlo (MCMC) with 4 chains of 5,000 iterations per chain, following a 200-sample burn-in. Convergence was assessed using the Gelman–Rubin R-hat statistic and visual inspection of trace plots (Gelman et al., 1995). If a model failed to converge, it was excluded from further consideration. Among models that successfully converged, model comparison was conducted using the Deviance Information Criterion (Spiegelhalter et al., 2002), with lower DIC values indicating better fit. In addition, a reduction in DIC greater than 5 was considered evidence of a substantial improvement in model fit, sufficient to justify increased model complexity (Tipples, 2018).

### Results

#### Analysis of task performance and subjective task experience

We first tested for load effects on performance and self-reported task perceptions as a manipulation check. Additionally, given that effort discounting is closely related to effort perception, we also examined whether the depression vs. nondepression groups differed in either task performance or their subjective experience of effort exertion. Prior research about this question has yielded mixed findings, but the general trend is that individuals with depression exhibit abnormal cognitive performance on effort-based decision making tasks (Barch et al., 2023; Horne et al., 2021).

To address these questions, we conducted linear mixed-effects model analyses examining the effects of effort level and group (depression vs. nondepression) on n-back task performance and subjective ratings measured by the NASA-TLX scale (see descriptive statistics in Tables 3 and 4). The models revealed significant effects of effort level across multiple measures, confirming that increasing cognitive load was associated with greater mental demand (b = 0.60, *p* <.001), subjective effort (b = 0.44, *p* =.003), frustration (b = 0.51, *p* <.001), and physical demand (b = 0.29, *p* =.005). Higher effort levels also led to lower estimated task performance (b =  − 0.78, *p* <.001), lower discriminability (d′) (b =  − 0.24, *p* <.001), reduced enjoyment (b =  − 0.30, *p* =.006), and longer response times (log-transformed RT; b = 0.024, *p* <.001). These findings validate the effectiveness of effort manipulation.

**Table 3:** Mean (SD) of task performance across effort levels by group in Experiment 3

	Nondepression group (N = 58)				Depression group (N = 50)			
Effort level	RT (M)	RT (SD)	d' (M)	d' (SD)	RT (M)	RT (SD)	d' (M)	d' (SD)
1	624.38	155.32	3.59	1.06	615.83	106.46	3.59	1.11
2	769.20	174.09	2.46	1.11	720.38	143.02	2.46	1.10
3	830.86	193.68	1.52	0.99	786.26	141.71	1.39	1.11
4	830.59	207.19	1.19	0.93	801.70	155.67	1.05	0.93
5	839.19	200.97	0.96	0.95	780.38	183.44	0.76	0.88
6	830.87	216.50	0.77	0.84	781.43	174.49	0.73	0.89

Effort level 1–6 represents 1-back to 6-back task. Performance metrics include d′ (sensitivity index) and mean response time (RT, ms). Linear mixed model analyses revealed significant linear effects of effort level on both d′ and RT (*p* <.001), with task performance declining as effort level increased

**Table 4:** Mean (SD) of subjective task experience across effort levels by group in Experiment 3

		Mental demand	Physical demand	Temporal demand	Estimated performance	Perceived effort	Frustration	Fun
Group	Effort level			Mean (SD)				
Nondepression group	1	8.03 (6.55)	3.84 (5.73)	4.86 (4.94)	16.98 (2.89)	8.1 (6.73)	2.38 (4.3)	12.81 (6.47)
	2	13.14 (4.99)	5.55 (6.32)	6.81 (5.08)	15 (3.63)	11.91 (5.55)	4.02 (4.9)	11.41 (6.08)
	3	14.93 (4.37)	5.84 (6.21)	8.24 (5.63)	12.22 (4.38)	14.34 (4.52)	5.57 (5.72)	10.21 (6.06)
	4	16.21 (3.27)	6.76 (6.92)	8.36 (5.66)	11.16 (4.47)	15.05 (3.8)	6.47 (6.21)	9.47 (6.1)
	5	16.14 (3.97)	6.81 (6.99)	9.57 (5.69)	10.53 (5.05)	15.45 (3.57)	6.91 (6.48)	9.16 (6.53)
	6	17 (3.61)	6.34 (6.7)	9.12 (6.02)	9.98 (5.03)	15.84 (4.03)	7.22 (6.41)	8.72 (5.79)
Depression group	1	9.56 (5.94)	4.12 (4.96)	7.56 (5.75)	16.6 (3.36)	9.98 (6.36)	7.94 (6.52)	8.84 (6.04)
	2	13.52 (4.14)	5.9 (5.72)	8.34 (5.03)	14.32 (4.42)	13.46 (4.01)	10.4 (5.74)	8.16 (5.65)
	3	15.84 (3.69)	6.9 (6.21)	9.66 (5.45)	11.02 (4.51)	15.42 (3.57)	12.42 (5.75)	7.34 (5.77)
	4	15.84 (3.78)	6.38 (5.87)	9.7 (5.61)	8.68 (5.19)	15.28 (3.72)	12.66 (5.45)	6.32 (5.82)
	5	16.56 (3.55)	6.44 (6.27)	9.66 (5.7)	7.72 (5.08)	14.98 (4.56)	13.72 (5.71)	5.22 (5.04)
	6	16.56 (3.91)	5.98 (6.17)	9.82 (5.46)	7.74 (5.28)	15.48 (4.09)	13.38 (6.04)	5.62 (5.6)

Effort level 1–6 represents 1-back to 6-back task. Subjective experiences were assessed after each n-back level using the NASA Task Load Index Scale (NASA-TLX), including mental demand, perceived effort, estimated performance, fun, frustration, temporal demand, and physical demand. Linear mixed model analyses revealed significant linear effects of effort level on all measures (*p* <.01) except for temporal demand: subjective workload ratings increased and perceived performance and fun decreased

Regarding group differences, the depressed individuals reported significantly lower enjoyment (b =  − 3.25, *p* =.006) and higher frustration (b = 5.76, *p* <.001) compared with nondepressed individuals. No significant group differences were found in objective task performance (d′, RT) or other subjective workload ratings when cognitive load was modeled as a continuous predictor. For perceived performance, there was no significant main effect of group in the primary analysis. However, in an exploratory analysis treating cognitive load as a categorical variable, the depression group reported significantly lower perceived performance than the nondepression group at higher effort levels (effort level 4: b =  − 2.475, *p* =.005; effort level 5: b =  − 2.814, *p* =.001; effort level 6: b =  − 2.243, *p* =.010). These findings indicate that although there were no significant group differences in objective task performance (d′, RT), the depression group experienced significantly less enjoyment and greater frustration compared with the nondepression group. Moreover, under higher effort levels, the depressed individuals tended to underestimate their own performance.

#### Linear mixed model analysis

Consistent with the analysis strategy in Experiment 1, a linear mixed-effects model was conducted to examine the effects of effort level, effort timing, and group (depression vs. nondepression) on SV. The final model included effort level (reference: 6-back), effort timing (reference: right now), group (reference: nondepression), and their interactions as fixed effects. To account for individual variability, random intercepts and random slopes for effort level by subject were included as random effects. Following previous research on cognitive effort discounting in depression (Westbrook et al., 2023), n-back performance (d′) was included as a covariate to control for task ability. Additionally, age, gender, and anxiety level (measured by GAD-7) were included as covariates to control for potential demographic and anxiety-related influences on effort valuation.

According to the fixed effect results, for the nondepression group, there was a temporal delay effect under the highest effort level: SV was significantly modulated by time to effort exertion, replicating the general pattern observed in Experiments 1 and 2. Specifically, SV significantly increased at all later timepoints compared with right now (*p*s < 0.01). Detailed model output is available in the supplementary materials (Table S3).

**Fig. 5:**
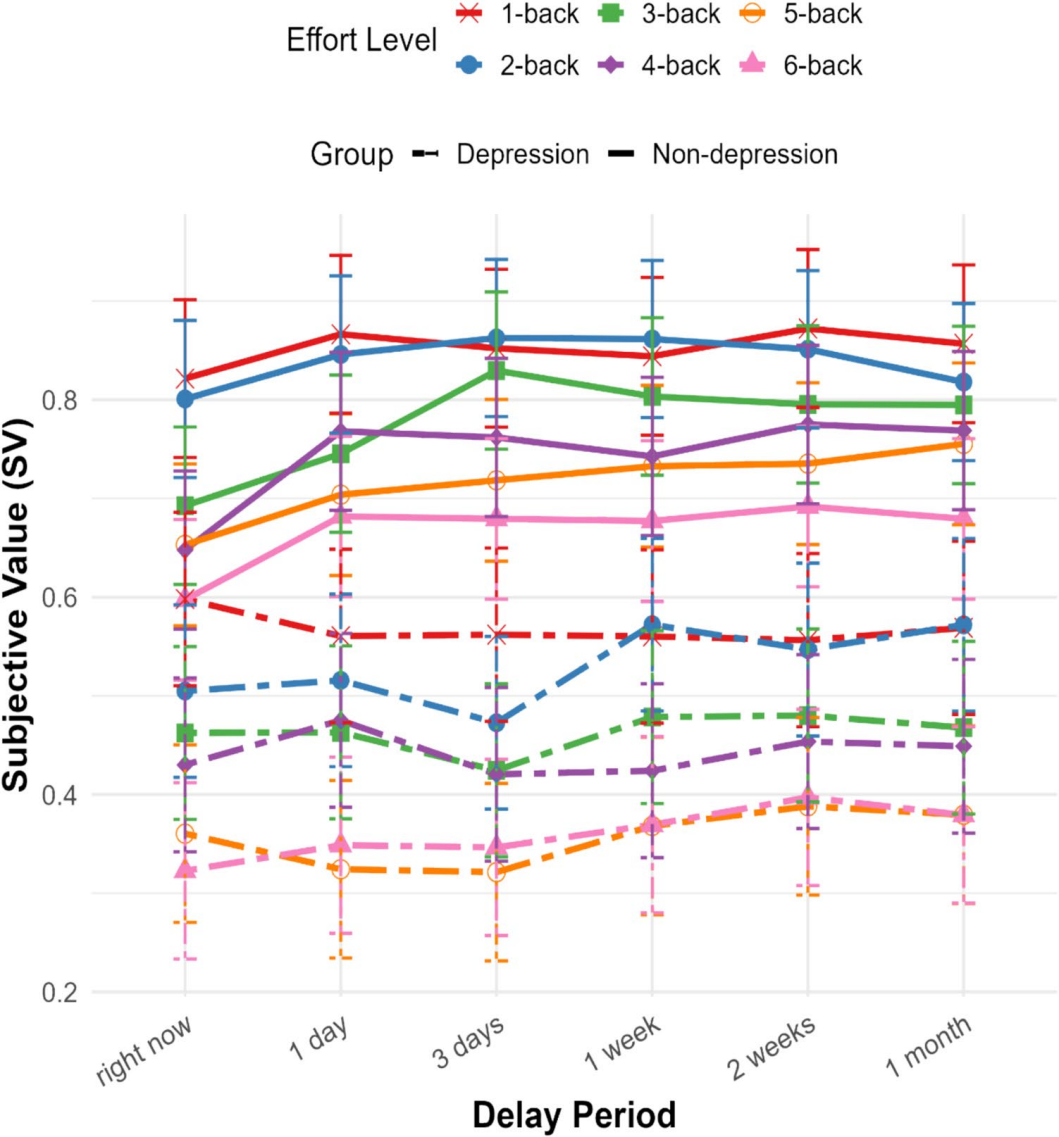
Subjective value (SV) across temporal delays in each effort level for depression and nondepression groups in Experiment 3. SV was measured across six temporal delays (X-axis: “right now” to “1 month”) and six effort levels (1-back to 6-back represents an increasing cognitive load, indicated by distinct colors and shapes). In the nondepression group (solid lines), SV increased with temporal delay at higher effort levels, indicating effort dependent temporal effort discounting effect. In contrast, the depression group (dashed lines) showed reduced temporal sensitivity, with SV remaining relatively flat across delays. Error bars represent ± 1 standard error

To explore further how SV changes across temporal delays by effort level within each group, we conducted pairwise comparisons. Temporal effects were modulated by both effort level and group (Fig. 5). For the nondepression group, SV increased over time delays, particularly at higher effort levels (3 to 6). Specifically, for effort levels 1 and 2, there were no significant temporal delay effects. By contrast, effort levels 3 to 6 exhibit a similar pattern whereby SV increases with delay. In particular, the 6-back task shows significant differences between SV for the present moment vs. all following delays (ps < 0.05). Further, the 5-back task shows significant differences between SV for the present moment and subsequent delays from 1 week to 1 month (*p*s < 0.05). The 4-back task shows significant differences between the present moment and all later delays (*p*s < 0.05), and the 3-back task shows significant differences between the present moment and delays ranging from 3 days to 1 month (*p*s < 0.01). Furthermore, the 3-back task showed a significant difference in SV between 1 and 3 days of delay. Notably, in all of these significant comparisons, the SV was consistently higher for doing the task later than sooner.

These results contrast with those of the depression group, for whom an effect of temporal delay was observed only for effort level 2 (i.e., 2-back). Concretely, the SV of performing the 2-back task after 3 days was significantly lower than doing the task both after 1 week (*p* = 0.007) and after 2 weeks (*p* = 0.008), which suggests greater effort discounting in the later period compared with before. Notably, individuals in the depression group showed significantly lower SV overall compared with the nondepression group (b = –0.313, *p* = 0.033), indicating heightened effort discounting among depressed participants.

#### HDDM analysis

Using the modeling procedure described above, the final selected model permitted drift rate (v) to vary according to group (depressed vs. nondepressed) and effort level (1-back to 6-back).

**Fig. 6:**
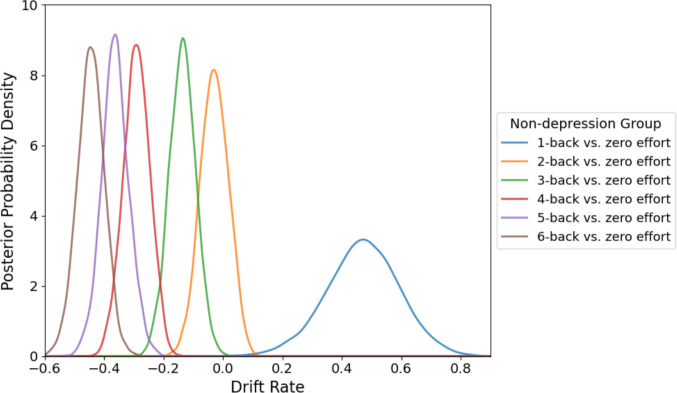
Posterior distributions for the drift rate parameter within each effort level for nondepression group in Experiment 3. Nondepression group showed positive drift rates at low effort levels, shifting to negative drift rates at higher effort levels, which indicates a transition from preferring the effortful choice to effort avoidance. *Note.* Positive drift rates indicate evidence accumulation favoring effortful choices, whereas negative values indicate evidence accumulation favoring zero-effort choices

Specifically, nondepressed individuals demonstrated high positive drift rates at low effort (1-back: M = 0.471, SD = 0.122), reflecting efficient evidence accumulation favoring the selection of the effortful option (Fig. 6). With increasing task demands, drift rates systematically decreased in magnitude, eventually becoming negative at higher effort levels. Significant reductions in drift rate were evident at elevated cognitive demands (3-back: β =  −.137, 95% CrI [− 0.226, − 0.051]; 4-back: β =  − 0.291, 95% CrI [− 0.381, − 0.208]; 5-back: β =  − 0.363, 95% CrI [− 0.454, − 0.271]; and 6-back: β =  − 0.445, 95% CrI [− 0.538, − 0.359]), indicating an adaptive shift toward effort avoidance as cognitive load intensified.

**Fig. 7:**
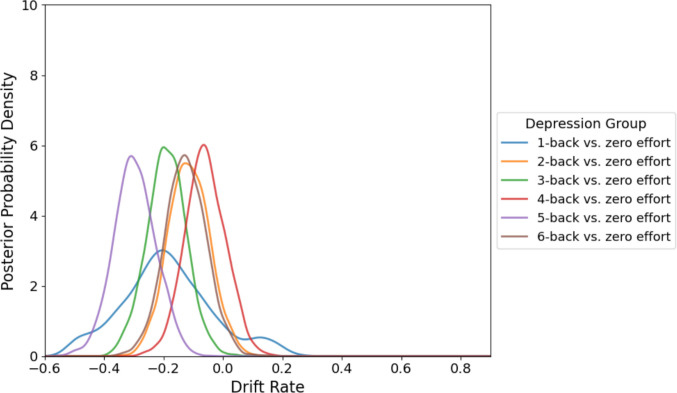
Posterior distributions for the drift rate parameter within each effort level for depression group in Experiment 3. The depression group showed consistently negative drift rates, reflecting a stable bias toward avoiding effort across all levels. *Note.* Positive drift rates indicate evidence accumulation favoring effortful choices, whereas negative values indicate evidence accumulation favoring zero-effort choices

Conversely, participants with depression exhibited a distinctly different pattern. Drift rates in this group were negative even at lower effort levels (1-back: M =  − 0.187, SD = 0.153; 2-back: M =  − 0.115, SD = 0.069) and remained consistently negative across increasing task demands (3-back: M =  − 0.190, SD = 0.066; 4-back: M =  − 0.060, SD = 0.068; 5-back: M =  − 0.297, SD = 0.071; 6-back: M =  − 0.126, SD = 0.070) (Fig. 7). However, no significant differences emerged across effort levels within the depressed group. These findings suggest a persistent and stable bias toward cognitive effort avoidance among individuals with depression.

Overall, these results illustrate that nondepressed participants adaptively modulate their decision-making processes according to task difficulty, whereas individuals with depression display generalized motivational disengagement.

#### Individual differences in effort valuation

As in Experiments 1 and 2, a correlation analysis was conducted between individual difference measures (e.g., NCS, subjective task experience) and SV. The results revealed a significant positive correlation between SV and subjectively estimated performance (r = 0.23, *p* = 0.015), suggesting that participants who experienced stronger self-efficacy on the task also discounted the effort less. However, in contrast to Experiments 1 and 2, this experiment did not reveal any significant correlations between perceived fun and SV. Nevertheless, a significant positive correlation between perceived fun and estimated performance (r = 0.366, *p* < 0.001) suggests that task enjoyment might still play an indirect role in shaping effort valuation.

### Discussion

Building on the results of Experiments 1 and 2, which demonstrated effort-dependent changes in SV across delays in the general population, Experiment 3 tested whether this pattern differs between individuals with and without depression. In the nondepression group, SV increased with delay at higher effort levels (particularly from 3-back to 6-back), suggesting a tendency to prefer deferring cognitively demanding tasks. In contrast, the depression group showed little to no delay-related increase in SV at higher effort levels, indicating a markedly different temporal valuation pattern. Notably, the depressed participants at the lowest effort level (1-back) and nondepressed participants at highest effort level (6-back) showed similar SV for the "right now" time point (SV =  ~.6; Fig. 5). However, only the nondepression group, but not the depression group, showed an increase in SV with delay. This dissociation between the groups suggests that the depression group’s flat SV trajectory across delay does not merely reflect a floor effect resulting from heightened task difficulty but instead indicates a fundamentally different way of allocating effort over time. Supporting this inference, HDDM analyses revealed that while nondepressed individuals flexibly shifted from positive to negative drift rates across increasing effort levels (reflecting effort-based strategic engagement and disengagement), the depression group exhibited consistently negative drift rates, consistent with a persistent avoidance of cognitive effort. Together, these results indicate that depression may involve a reduced or rigid capacity to update value over time in response to cognitive demands.

## General discussion

### Cognitive load moderates temporal effort discounting

The current study investigated the temporal dynamics of effort discounting across varying cognitive loads and explored its link to depression-related differences. Using two distinct cognitive effort tasks (backward typing in Experiment 1 and n-back in Experiments 2 and 3) in combination with the COG-ED paradigm, we found clear evidence that cognitive load moderates temporal effort discounting, and that this modulation is sensitive to depression levels.

Toward this end, we built on previous work by implementing several task features that isolate the effect of temporal delay on valuation of cognitive effort. In particular, monetary rewards for all choices were fixed for delivery 1 month postexperiment, which eliminates confounds due to reward delay discounting. That is, participants received their rewards 1 month after the experiment irrespective of whether or not they chose to do the effortful tasks. We also increased the number of timepoints to six (from “immediate” to “1 month”) to examine finer fluctuations in SV with time. Using this methodology, we consistently observed a strong interaction between effort level and time delay.

A key observation across studies was that delay increased subjective value only under high effort demands. Interestingly, even when using the same backward typing task as prior work (Johnson & Most, 2023), we found no delay effect at their highest effort level (100 words), nor at a higher level (150 words). The delay effect only emerged at the most aversive load (200 words). This suggests that preferences to defer effort may arise only when task demands are sufficiently high to trigger avoidance. One plausible reason is the use of real incentives: when faced with actual monetary consequences, participants may be less willing to give up value simply to delay mild effort, resulting in uniformly high subjective values at lower effort levels—a ceiling effect that further suppresses delay sensitivity under low load. In contrast, only when the cognitive burden is high enough does the cost of immediate effort outweigh the reward, prompting a preference for delay. Thus, real stakes may sharpen the threshold at which effort is seen as worth deferring.

Noticeably, in our study, temporal delay effects were primarily observed between the immediate and all later timepoints, while differences among the future delays (e.g., between 3 days and 1 month) were minimal. This finding aligns with prior work showing significant differences between present effort and future effort, but no difference between 1 day and 1 month (Johnson & Most, 2023). A likely explanation is that mental simulations of future effort are uniformly incomplete, resulting in reduced subjective effort costs irrespective of the precise temporal proximity (Gilbert & Wilson, 2007). Apart from that possibility, both in our study and in prior work (Johnson & Most, 2023), all of the delays were varied within the near future (i.e., within 1 month), and the effort tasks were relatively brief in duration (i.e., up to 40 min in Experiment 1 and up to 15 min in Experiments 2 and 3). As such, the participants might have prioritized avoiding immediate effort rather than distinguishing between delays in the relatively near-future. This consistent avoidance of immediate effort also helps to rule out a potential alternative explanation—that participants preferred to complete the task right away simply to “get it over with,” given that they were already engaged in the study. If that were the case, we would have expected a preference for immediate effort. Instead, the opposite pattern emerged. Moreover, because the study was conducted online, the inconvenience of returning to complete the study was minimal, making it less likely that participants chose the immediate option based on convenience.

In sum, our results show that effort discounting over time is load-dependent: high-effort tasks become less costly when delayed, while low-effort tasks are valued similarly across time. This adaptive delay preference under high load underscores the dynamic nature of cognitive effort costs and their dependence on both task difficulty and temporal delay. These findings offer novel insights into preference reversal and procrastination. Specifically, our results suggest that when making decisions about future high-effort tasks, individuals tend to undervalue the subjective cost of future effort, making delayed options more appealing. However, when that future effort becomes imminent, its subjective cost increases, leading to a reversal of preference—a shift toward postponing the task again. This temporal distortion in evaluating cognitive effort likely contributes to procrastination, particularly for cognitively demanding tasks, where the steep discounting of immediate effort encourages individuals to repeatedly defer action. These findings suggest that procrastination may not only reflect delay discounting of rewards, as traditionally framed (Zentall, 2021; Zhang & Ma, 2024), but may also stem from dynamic inconsistency in effort-related decisions. That is, people discount cognitive effort less when it lies in the future, which may drive them to postpone harder tasks to a later time. This nuanced view helps explain common self-regulatory failures and highlights the potential role of effort discounting dynamics in shaping goal-directed behavior over time.

### Depression-related differences in temporal effort discounting

Experiment 3 replicated the load-dependent temporal discounting pattern observed in Experiment 2 and revealed distinct behavioral signatures in individuals with depression. Consistent with previous research, both groups showed a main effect of effort level on effort discounting (Apps et al., 2015; Westbrook et al., 2013), and depressed individuals discounted cognitive effort more steeply than nondepressed individuals (Ang et al., 2023). Crucially, time-delay effects diverged across groups: whereas nondepressed participants preferred to delay high-effort tasks (notably from the 3-back to 6-back levels), depressed individuals only showed a delay effect at a moderate (2-back) level. Furthermore, even when the SVs at the immediate time point were matched between groups (i.e., low effort (1-back) for depressed vs. high effort (6-back) for nondepressed, SV =  ~.6), only the nondepression group showed increased SV with delay. This pattern suggests that depression impairs the integration of temporal information into value-based decision-making. Although the nondepressed individuals flexibly adapt their valuation of the task incentives based on both the task difficulty and its timing (e.g., “I’d rather do this hard task later”), the depressed individuals exhibit a flatter response to delay (e.g., “I don’t want to do this task, no matter when”). This reduced temporal sensitivity may contribute to persistent effort avoidance commonly seen in depression.

To further explore group differences in decision dynamics, we conducted hierarchical drift diffusion modeling (HDDM). Results showed that in the nondepression group, the drift rates were generally positive at low effort levels and became negative as the task demands increased, consistent with a flexible, load-sensitive mechanisms for evidence accumulation. In contrast, the depression group exhibited consistently negative drift rates across effort levels, suggesting a stable bias toward effort avoidance regardless of task difficulty. Although the delay factor was not included in the model due to convergence issues, these results align with the behavioral findings by supporting the notion that individuals with depression may exhibit reduced flexibility in evaluating effort costs. The depression group appeared less responsive to changes in task difficulty and, as shown behaviorally, also showed less modulation by temporal delay. Together, the findings suggest that depression may involve a more rigid decision-making process marked by elevated and persistent effort aversion.

In addition to the primary factors examined in this study, effort level and effort timing, our supplementary analyses revealed that perceived fun and subjective performance estimates were positively correlated with the average SV assigned to effort-reward options. These findings suggest that task enjoyment and self-efficacy beliefs (i.e., confidence in one's ability to perform well) play a role in how individuals evaluate effortful tasks. This aligns with existing literature indicating that intrinsic motivation enhances engagement and valuation of effortful activities (Deci & Ryan, 1985; Mlynski et al., 2025), and that higher self-efficacy is associated with greater willingness to exert effort and persevere (Bandura, 1997). Moreover, perceptions of self-agency—the belief that one has control over task outcomes—have been shown to increase both task value and effort investment (Moscarello & Hartley, 2017).

Recent work further supports the idea that effort is not always experienced as a cost, but can increase the perceived value of activities under certain psychological and contextual conditions. For instance, Campbell et al. (2025) found that effortful leisure activities, such as puzzle-solving or skill-based hobbies, are perceived as more meaningful despite being less immediately enjoyable—indicating a trade-off between hedonic and eudaimonic value. Similarly, Inzlicht et al. (2025) propose that the so-called “effort paradox” may be resolved by recognizing effort’s dual role as both an aversive cost and a source of intrinsic reward, depending on how outcomes are attributed and how culturally effort is framed. Moreover, context plays a crucial role, as Embrey et al. (2024) demonstrated that individuals are more willing to choose cognitively demanding tasks when the alternative is boredom, and that structured environments (e.g., lab vs. online) significantly increase preference for high-effort options.

Building on this, these findings underscore the importance of task-specific affective and contextual factors in shaping effort valuation, particularly in contrast to more stable trait-level constructs. Notably, while several studies have reported a positive association between NCS scores and SV, the broader literature presents mixed findings. For instance, although Westbrook et al. (2013) found a positive NCS–SV correlation, subsequent studies failed to replicate this effect or observed that it disappeared after controlling for task performance (Crawford et al., 2021; Kramer et al., 2021). Zerna et al. (2023) further demonstrated that NCS-related differences in SV only emerged at higher task loads, suggesting that this relationshi*p* may be task-dependent rather than consistent across contexts. In our study, NCS was not significantly associated with SV, whereas perceived task enjoyment was positively correlated with SV in the general population, as shown in the first two experiments. This potential dissociation between trait-level cognitive motivation (e.g., NCS) and task-specific affective engagement (e.g., enjoyment) highlights a promising direction for future research. Future studies could examine whether task enjoyment mediates the relationshi*p* between NCS and SV.

### Limitation and future directions

These results suggest several avenues for future research. First, although our design effectively addressed the core aim of the study—to examine temporal effort discounting—it remains an important question whether variations in reward magnitude might influence this effect. Specifically, larger or smaller rewards may moderate temporal effort discounting patterns, particularly in depressed individuals, who often exhibit reduced reward sensitivity (Umemoto & Holroyd, 2017). It is plausible that larger incentives could mitigate effort aversion in depressed individuals, leading to more flexible effort discounting patterns that resemble those of nondepressed individuals. Future research could explore how reward magnitude interacts with cognitive load and effort timing, especially in clinical populations, to better understand motivational dynamics.

Second, under the assumption that the participants would likely be more available to do the task in the far future than in the near future, and thus base their choices on time availability rather than on expected effort, the experiment was designed to minimize this potential cofound. In particular, we allowed participants a two-day window to complete their selected task such that, for example, a task selected for “in 1 day” could be completed at any point within the following two days. Nevertheless, it remains possible that participants interpreted the timepoints literally and overlooked this flexibility when making their decisions. As a result, choices to delay effort may have been driven more by momentary fatigue or perceived burden, especially for choices associated with “immediate” effort, rather than a deliberate evaluation of temporal delay (Müller et al., 2021). To better control for fatigue-related influences, future research could, for example, separate the practice phase from the decision phase over different days, or adjust the temporal delay options by comparing short delays (e.g., “tomorrow”) with longer ones (e.g., “next week” or “in a month”). These adjustments would help clarify whether participants’ preferences for delaying effort reflect genuine temporal discounting or strategic avoidance due to transient fatigue.

Additionally, future research could examine how neural mechanisms shape temporal effort discounting, particularly the role of the anterior cingulate cortex (ACC), which plays a central role in motivating effortful behavior (Holroyd & McClure, 2015; Holroyd & Umemoto, 2016; Holroyd & Yeung, 2012; Massar et al., 2015). Notably, a computational model of ACC function during effortful decision making requires preference reversal of the sort exhibited here; in these simulations, ACC is responsible for sustaining goal-directed behaviors when increasing effort costs conflict with the original choice to approach the goal (Holroyd & McClure, 2015). Consistent with this view, Seamans et al. (2024) demonstrated that ACC ensembles dynamically track the value of effortful options in delay discounting tasks, suggesting a key role of ACC in encoding subjective effort costs over time​. Similarly, Müller et al. (2021) demonstrated that medial prefrontal and ACC regions integrate fatigue signals into effort valuation, with accumulated fatigue increasing effort aversion and reducing persistence.​ Chong and colleagues (2016) also highlighted the ACC’s role in integrating effort and reward during decision-making and showed that reduced ACC engagement is linked to greater effort aversion and motivational deficits. Further, they noted that trait persistence is associated with stronger ACC engagement during effort-based decisions, indicating a link between persistence and neural tolerance of effort. Together, these findings highlight the ACC as a promising target for future research into the neural basis of temporal effort discounting.

Building on these directions, future work could also consider integrating both temporal effort discounting and temporal reward discounting within a within-subject design, as demonstrated in earlier work using similar paradigms (Westbrook et al., 2013). While our current study was deliberately designed to isolate the effect of effort timing by holding reward timing constant, examining both types of discounting within the same participants could offer important insights into how temporal preferences for effort and reward interact. Such a design would further enable researchers to test whether individual differences in delay sensitivity generalize across reward- and effort-based domains, and whether shared or distinct cognitive and neural mechanisms support these processes.

Finally, these findings suggest new avenues for precision psychiatry (Fernandes et al., 2017), particularly by distinguishing between different types of motivational deficits across clinical conditions. In particular, although depression, the negative symptoms of schizophrenia, apathy and ADHD all involve effort aversion (Ang et al., 2023; Barch et al., 2023; Holroyd & Umemoto, 2016; Le Bouc et al., 2023; Orhan et al., 2023), the temporal patterns of effort discounting might differ between populations. The reduced temporal sensitivity observed in our depressed sample, who were insensitive to delays in effort execution even for high effort levels, could help differentiate depression from other disorders such as schizophrenia-related anhedonia or ADHD, where effort-reward computations could involve different neural mechanisms. By characterizing temporal effort discounting profiles across clinical groups, future research could improve diagnostic specificity and inform personalized interventions. For example, patients who abnormally discount effort costs over time might benefit from direct reduction of perceived effort costs, whereas others might respond better to time-management strategies. Incorporating temporal effort discounting tasks into clinical assessments aligns with precision psychiatry’s goal to tailor treatments based on behavioral phenotypes.

### Conclusions

The present study reveals that cognitive load moderates temporal effort discounting: under high effort, individuals value delayed effort more, while under low effort, timing has little effect. However, depressed individuals show heightened effort aversion, with delay offering minimal benefit overall, indicating reduced temporal flexibility in effort valuation. These findings highlight the interplay between cognitive load, effort timing, and depression-related differences in shaping motivation.

## Supplementary Information

Below is the link to the electronic supplementary material.

Supplementary file1 (DOCX 115 KB)

## Data Availability

The data for the analyses reported in the current study are available at https://osf.io/z3yv4/.

## Declarations

### Open practices statement

The data for the analyses reported in the current study are available at https://osf.io/z3yv4/.

None of the experiments were preregistered.
