# Supplementary material for: Temporal dynamics of effort discounting: The roles of cognitive load and depression
Source: Cogn Affect Behav Neurosci. 2026 Feb 14;26(2):449–68. doi: 10.3758/s13415-026-01410-8 (PMC13095905; doi:10.3758/s13415-026-01410-8)
Supplement: Supplementary file 1 — Supplementary file1 (DOCX 115 KB) [file 13415_2026_1410_MOESM1_ESM.docx]

**Experiment 1**

| **Predictors** | **b** | **SE** | **conf.low** | **conf.high** | **p** |
| --- | --- | --- | --- | --- | --- |
| (Intercept) | 0.557 | 0.048 | 0.462 | 0.653 | **0.000** |
| effort level1 | 0.295 | 0.034 | 0.229 | 0.361 | **0.000** |
| effort level2 | 0.170 | 0.034 | 0.104 | 0.236 | **0.000** |
| effort level3 | 0.078 | 0.034 | 0.011 | 0.144 | **0.022** |
| timepoint2 | 0.015 | 0.034 | -0.051 | 0.081 | 0.656 |
| timepoint3 | 0.000 | 0.034 | -0.066 | 0.066 | 1.000 |
| timepoint4 | 0.030 | 0.034 | -0.036 | 0.096 | 0.374 |
| timepoint5 | 0.060 | 0.034 | -0.006 | 0.126 | 0.075 |
| timepoint6 | 0.098 | 0.034 | 0.031 | 0.164 | **0.004** |
| effort level1:timepoint2 | -0.015 | 0.048 | -0.109 | 0.079 | 0.753 |
| effort level2:timepoint2 | 0.012 | 0.048 | -0.081 | 0.106 | 0.793 |
| effort level3:timepoint2 | 0.032 | 0.048 | -0.061 | 0.126 | 0.496 |
| effort level1:timepoint3 | 0.007 | 0.048 | -0.086 | 0.101 | 0.875 |
| effort level2:timepoint3 | 0.005 | 0.048 | -0.089 | 0.099 | 0.917 |
| effort level3:timepoint3 | 0.015 | 0.048 | -0.079 | 0.109 | 0.753 |
| effort level1:timepoint4 | -0.048 | 0.048 | -0.141 | 0.046 | 0.319 |
| effort level2:timepoint4 | 0.015 | 0.048 | -0.079 | 0.109 | 0.753 |
| effort level3:timepoint4 | 0.025 | 0.048 | -0.069 | 0.119 | 0.600 |
| effort level1:timepoint5 | -0.088 | 0.048 | -0.181 | 0.006 | 0.067 |
| effort level2:timepoint5 | -0.063 | 0.048 | -0.156 | 0.031 | 0.190 |
| effort level3:timepoint5 | -0.015 | 0.048 | -0.109 | 0.079 | 0.753 |
| effort level1:timepoint6 | -0.093 | 0.048 | -0.186 | 0.001 | 0.053 |
| effort level2:timepoint6 | -0.103 | 0.048 | -0.196 | -0.009 | **0.032** |
| effort level3:timepoint6 | -0.058 | 0.048 | -0.151 | 0.036 | 0.228 |

**Table S1. Regression of subjective values on effort level and effort timing variables in Experiment 1** Effort level 4 and Timepoint 1 served as reference levels in treatment contrast coding. Effort level 1–4 correspond to increasing levels of cognitive load in backwards typing task (effort level 1: 50-word; effort level 2: 100-word; effort level 3: 150-word; effort level 4: 200-word); Timepoints 1–6 represent increasing delays in effort exertion (timepoint 1: right now; timepoint 2: in 1 day; timepoint 3: in 3 days; timepoint 4: in 1 week; timepoint 5: in 2 weeks; timepoint 6: in 1 month). Estimates reflect the change in SV relative to the reference levels; positive estimates indicate higher SV. The model included random intercepts for subjects as a random effect. Bold p-values indicate p < .05.

**Experiment 2**

|  | **Predictors** | **b** | **SE** |  |  | **conf.low** | **conf.high** |  |  |  | **p** |
| --- | --- | --- | --- | --- | --- | --- | --- | --- | --- | --- | --- |
|  | (Intercept) | 0.603 | 0.054 |  |  | 0.493 | 0.712 |  |  |  | **0.000** |
|  | effort level1 | 0.222 | 0.048 |  |  | 0.126 | 0.318 |  |  |  | **0.000** |
|  | effort level2 | 0.239 | 0.047 |  |  | 0.145 | 0.332 |  |  |  | **0.000** |
|  | effort level3 | 0.188 | 0.038 |  |  | 0.113 | 0.263 |  |  |  | **0.000** |
|  | effort level4 | 0.065 | 0.034 |  |  | -0.002 | 0.133 |  |  |  | 0.058 |
|  | effort level5 | 0.022 | 0.030 |  |  | -0.037 | 0.081 |  |  |  | 0.458 |
|  | timepoint2 | 0.038 | 0.025 |  |  | -0.011 | 0.088 |  |  |  | 0.129 |
|  | timepoint3 | 0.040 | 0.025 |  |  | -0.009 | 0.090 |  |  |  | 0.111 |
|  | timepoint4 | 0.090 | 0.025 |  |  | 0.040 | 0.139 |  |  |  | **0.000** |
|  | timepoint5 | 0.077 | 0.025 |  |  | 0.027 | 0.126 |  |  |  | **0.002** |
|  | timepoint6 | 0.056 | 0.025 |  |  | 0.007 | 0.106 |  |  |  | **0.026** |
|  | N-back performance(d’) | -0.004 | 0.035 |  |  | -0.074 | 0.066 |  |  |  | 0.911 |
|  | effort level1:timepoint2 | -0.011 | 0.036 |  |  | -0.081 | 0.059 |  |  |  | 0.760 |
|  | effort level2:timepoint2 | -0.024 | 0.036 |  |  | -0.094 | 0.046 |  |  |  | 0.501 |
|  | effort level3:timepoint2 | -0.040 | 0.036 |  |  | -0.110 | 0.030 |  |  |  | 0.261 |
|  | effort level4:timepoint2 | -0.027 | 0.036 |  |  | -0.097 | 0.043 |  |  |  | 0.455 |
|  | effort level5:timepoint2 | 0.031 | 0.036 |  |  | -0.039 | 0.101 |  |  |  | 0.387 |
|  | effort level1:timepoint3 | 0.006 | 0.036 |  |  | -0.064 | 0.076 |  |  |  | 0.869 |
|  | effort level2:timepoint3 | -0.021 | 0.036 |  |  | -0.091 | 0.049 |  |  |  | 0.554 |
|  | effort level3:timepoint3 | -0.011 | 0.036 |  |  | -0.081 | 0.059 |  |  |  | 0.765 |
|  | effort level4:timepoint3 | 0.005 | 0.036 |  |  | -0.065 | 0.075 |  |  |  | 0.893 |
|  | effort level5:timepoint3 | 0.041 | 0.036 |  |  | -0.029 | 0.111 |  |  |  | 0.249 |
|  | effort level1:timepoint4 | -0.027 | 0.036 |  |  | -0.097 | 0.043 |  |  |  | 0.451 |
|  | effort level2:timepoint4 | -0.074 | 0.036 |  |  | -0.144 | -0.004 |  |  |  | **0.039** |
|  | effort level3:timepoint4 | -0.082 | 0.036 |  |  | -0.152 | -0.012 |  |  |  | **0.022** |
|  | effort level4:timepoint4 | -0.052 | 0.036 |  |  | -0.122 | 0.018 |  |  |  | 0.146 |
|  | effort level5:timepoint4 | -0.077 | 0.036 |  |  | -0.147 | -0.007 |  |  |  | **0.031** |
|  | effort level1:timepoint5 | -0.048 | 0.036 |  |  | -0.118 | 0.022 |  |  |  | 0.177 |
|  | effort level2:timepoint5 | -0.060 | 0.036 |  |  | -0.130 | 0.010 |  |  |  | 0.091 |
|  | effort level3:timepoint5 | -0.070 | 0.036 |  |  | -0.140 | 0.000 |  |  |  | 0.051 |
|  | effort level4:timepoint5 | -0.024 | 0.036 |  |  | -0.094 | 0.046 |  |  |  | 0.503 |
|  | effort level5:timepoint5 | -0.025 | 0.036 |  |  | -0.095 | 0.045 |  |  |  | 0.489 |
|  | effort level1:timepoint6 | -0.005 | 0.036 |  |  | -0.075 | 0.065 |  |  |  | 0.879 |
|  | effort level2:timepoint6 | -0.047 | 0.036 |  |  | -0.117 | 0.023 |  |  |  | 0.189 |
|  | effort level3:timepoint6 | -0.044 | 0.036 |  |  | -0.114 | 0.026 |  |  |  | 0.219 |
|  | effort level4:timepoint6 | -0.003 | 0.036 |  |  | -0.073 | 0.067 |  |  |  | 0.923 |
|  | effort level5:timepoint6 | 0.015 | 0.036 |  |  | -0.055 | 0.085 |  |  |  | 0.681 |

**Table S2. Regression of subjective values on effort level and effort timing variables in Experiment 2** Effort level 6 and Timepoint 1 served as reference levels in treatment contrast coding. Effort levels corresponded to task difficulty in the N-back task, ranging from 1-back (effort level 1) to 6-back (effort level 6); Timepoints 1–6 represent increasing delays in effort exertion (timepoint 1: right now; timepoint 2: in 1 day; timepoint 3: in 3 days; timepoint 4: in 1 week; timepoint 5: in 2 weeks; timepoint 6: in 1 month). Estimates reflect the change in SV relative to the reference levels; positive estimates indicate higher SV. The model included random intercepts and random slopes for effort level by subjects as random effects. Bold p-values indicate p < .05.

**Experiment 3**

**Correlation analysis**

A spearman correlation analysis revealed significant associations between measures of depression (PHQ-9, CES-D), anxiety (GAD-7), anhedonia (SHAPS), need for cognition (NCS), and procrastination (PPS)(Fig. S1). As expected, PHQ-9 and CES-D were highly correlated (r = 0.90, p < 0.001), indicating strong agreement between these measures of depressive symptoms. Similarly, GAD-7 showed strong positive correlations with both PHQ-9 (r = 0.90, p < 0.001) and CES-D (r = 0.90, p < 0.001), supporting that anxiety is a comorbidity of depression disorder indicated by previous studies(Choi et al., 2020). SHAPS, a measure of anhedonia, which is a core symptom of depression, was significantly correlated with PHQ-9 (r = 0.58, p < 0.001), CES-D (r = 0.60, p < 0.001), and GAD-7 (r = 0.59, p < 0.001).

Procrastination (PPS) was positively correlated with PHQ-9 (r = 0.67, p < 0.001), CES-D (r = 0.64, p < 0.001), SHAPS (r = 0.58, p < 0.001), and GAD-7 (r = 0.70, p < 0.001), indicating that greater procrastination tendencies were linked to higher levels of depression, anxiety, and anhedonia. Conversely, need for cognition (NCS) was negatively correlated with PHQ-9 (r = -0.31, p = 0.001), CES-D (r = -0.29, p = 0.002), GAD-7 (r = -0.27, p = 0.004), and PPS (r = -0.32, p < 0.001), suggesting that individuals who enjoy engaging in cognitively demanding activities more tend to report lower depressive symptoms, anxiety, and procrastination. Interestingly, NCS was not significantly correlated with SHAPS (r = -0.08, p = 0.36), indicating that need for cognition may be relatively independent of anhedonia.


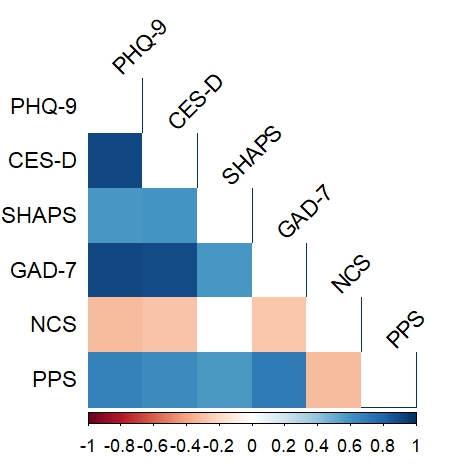


Fig S1. Correlation among questionnaire variables

|  | **Predictors** | **b** | **SE** |  | **conf.low** | **conf.high** |  | **p.value** |
| --- | --- | --- | --- | --- | --- | --- | --- | --- |
|  | (Intercept) | 0.606 | 0.096 |  | 0.416 | 0.796 |  | **0.000** |
|  | effort level1 | 0.224 | 0.046 |  | 0.134 | 0.314 |  | **0.000** |
|  | effort level2 | 0.203 | 0.043 |  | 0.119 | 0.288 |  | **0.000** |
|  | effort level3 | 0.095 | 0.041 |  | 0.014 | 0.176 |  | **0.021** |
|  | effort level4 | 0.050 | 0.035 |  | -0.018 | 0.119 |  | 0.150 |
|  | effort level5 | 0.056 | 0.031 |  | -0.006 | 0.117 |  | 0.076 |
|  | timepoint2 | 0.084 | 0.027 |  | 0.032 | 0.137 |  | **0.002** |
|  | timepoint3 | 0.082 | 0.027 |  | 0.030 | 0.134 |  | **0.002** |
|  | timepoint4 | 0.080 | 0.027 |  | 0.027 | 0.132 |  | **0.003** |
|  | timepoint5 | 0.094 | 0.027 |  | 0.042 | 0.147 |  | **0.000** |
|  | timepoint6 | 0.082 | 0.027 |  | 0.030 | 0.134 |  | **0.002** |
|  | depression group | -0.275 | 0.153 |  | -0.578 | 0.028 |  | 0.075 |
|  | age | -0.008 | 0.031 |  | -0.070 | 0.055 |  | 0.807 |
|  | Male vs. Female | -0.016 | 0.066 |  | -0.147 | 0.115 |  | 0.804 |
|  | N-back performance(d’) | 0.062 | 0.031 |  | -0.000 | 0.124 |  | 0.050 |
|  | GAD-7 | 0.117 | 0.074 |  | -0.031 | 0.264 |  | 0.120 |
|  | effort level1:timepoint2 | -0.040 | 0.038 |  | -0.114 | 0.035 |  | 0.296 |
|  | effort level2:timepoint2 | -0.039 | 0.038 |  | -0.114 | 0.035 |  | 0.298 |
|  | effort level3:timepoint2 | -0.032 | 0.038 |  | -0.106 | 0.042 |  | 0.402 |
|  | effort level4:timepoint2 | 0.036 | 0.038 |  | -0.038 | 0.110 |  | 0.344 |
|  | effort level5:timepoint2 | -0.034 | 0.038 |  | -0.108 | 0.041 |  | 0.375 |
|  | effort level1:timepoint3 | -0.051 | 0.038 |  | -0.126 | 0.023 |  | 0.175 |
|  | effort level2:timepoint3 | -0.020 | 0.038 |  | -0.094 | 0.054 |  | 0.594 |
|  | effort level3:timepoint3 | 0.055 | 0.038 |  | -0.019 | 0.129 |  | 0.146 |
|  | effort level4:timepoint3 | 0.032 | 0.038 |  | -0.042 | 0.106 |  | 0.396 |
|  | effort level5:timepoint3 | -0.017 | 0.038 |  | -0.091 | 0.058 |  | 0.662 |
|  | effort level1:timepoint4 | -0.057 | 0.038 |  | -0.131 | 0.017 |  | 0.131 |
|  | effort level2:timepoint4 | -0.019 | 0.038 |  | -0.093 | 0.055 |  | 0.615 |
|  | effort level3:timepoint4 | 0.031 | 0.038 |  | -0.043 | 0.105 |  | 0.414 |
|  | effort level4:timepoint4 | 0.015 | 0.038 |  | -0.059 | 0.089 |  | 0.690 |
|  | effort level5:timepoint4 | -0.000 | 0.038 |  | -0.074 | 0.074 |  | 0.996 |
|  | effort level1:timepoint5 | -0.044 | 0.038 |  | -0.118 | 0.030 |  | 0.247 |
|  | effort level2:timepoint5 | -0.044 | 0.038 |  | -0.118 | 0.030 |  | 0.245 |
|  | effort level3:timepoint5 | 0.008 | 0.038 |  | -0.066 | 0.082 |  | 0.829 |
|  | effort level4:timepoint5 | 0.033 | 0.038 |  | -0.041 | 0.107 |  | 0.387 |
|  | effort level5:timepoint5 | -0.012 | 0.038 |  | -0.086 | 0.062 |  | 0.748 |
|  | effort level1:timepoint6 | -0.047 | 0.038 |  | -0.121 | 0.027 |  | 0.217 |
|  | effort level2:timepoint6 | -0.065 | 0.038 |  | -0.139 | 0.009 |  | 0.087 |
|  | effort level3:timepoint6 | 0.020 | 0.038 |  | -0.054 | 0.094 |  | 0.594 |
|  | effort level4:timepoint6 | 0.039 | 0.038 |  | -0.035 | 0.113 |  | 0.302 |
|  | effort level5:timepoint6 | 0.020 | 0.038 |  | -0.054 | 0.095 |  | 0.591 |
|  | effort level1:depression group | 0.051 | 0.067 |  | -0.081 | 0.184 |  | 0.447 |
|  | effort level2:depression group | -0.021 | 0.063 |  | -0.145 | 0.102 |  | 0.735 |
|  | effort level3:depression group | 0.044 | 0.060 |  | -0.075 | 0.163 |  | 0.463 |
|  | effort level4:depression group | 0.057 | 0.051 |  | -0.044 | 0.158 |  | 0.266 |
|  | effort level5:depression group | -0.018 | 0.046 |  | -0.108 | 0.072 |  | 0.697 |
|  | timepoint2:depression group | -0.058 | 0.039 |  | -0.136 | 0.019 |  | 0.137 |
|  | timepoint3:depression group | -0.058 | 0.039 |  | -0.135 | 0.019 |  | 0.139 |
|  | timepoint4:depression group | -0.033 | 0.039 |  | -0.110 | 0.044 |  | 0.400 |
|  | timepoint5:depression group | -0.020 | 0.039 |  | -0.097 | 0.057 |  | 0.610 |
|  | timepoint6:depression group | -0.025 | 0.039 |  | -0.103 | 0.052 |  | 0.519 |
|  | effort level1:timepoint2:depression group | -0.024 | 0.056 |  | -0.133 | 0.085 |  | 0.667 |
|  | effort level2:timepoint2:depression group | 0.024 | 0.056 |  | -0.085 | 0.133 |  | 0.664 |
|  | effort level3:timepoint2:depression group | 0.006 | 0.056 |  | -0.103 | 0.115 |  | 0.910 |
|  | effort level4:timepoint2:depression group | -0.017 | 0.056 |  | -0.126 | 0.092 |  | 0.763 |
|  | effort level5:timepoint2:depression group | -0.028 | 0.056 |  | -0.138 | 0.081 |  | 0.609 |
|  | effort level1:timepoint3:depression group | -0.009 | 0.056 |  | -0.118 | 0.100 |  | 0.877 |
|  | effort level2:timepoint3:depression group | -0.036 | 0.056 |  | -0.145 | 0.073 |  | 0.520 |
|  | effort level3:timepoint3:depression group | -0.117 | 0.056 |  | -0.226 | -0.008 |  | **0.036** |
|  | effort level4:timepoint3:depression group | -0.066 | 0.056 |  | -0.175 | 0.044 |  | 0.239 |
|  | effort level5:timepoint3:depression group | -0.046 | 0.056 |  | -0.155 | 0.063 |  | 0.406 |
|  | effort level1:timepoint4:depression group | -0.027 | 0.056 |  | -0.136 | 0.082 |  | 0.623 |
|  | effort level2:timepoint4:depression group | 0.040 | 0.056 |  | -0.069 | 0.149 |  | 0.476 |
|  | effort level3:timepoint4:depression group | -0.062 | 0.056 |  | -0.171 | 0.048 |  | 0.269 |
|  | effort level4:timepoint4:depression group | -0.068 | 0.056 |  | -0.177 | 0.041 |  | 0.223 |
|  | effort level5:timepoint4:depression group | -0.039 | 0.056 |  | -0.148 | 0.070 |  | 0.486 |
|  | effort level1:timepoint5:depression group | -0.072 | 0.056 |  | -0.181 | 0.037 |  | 0.194 |
|  | effort level2:timepoint5:depression group | 0.012 | 0.056 |  | -0.097 | 0.121 |  | 0.833 |
|  | Effort level3:timepoint5:depression group | -0.065 | 0.056 |  | -0.174 | 0.044 |  | 0.244 |
|  | effort level4:timepoint5:depression group | -0.084 | 0.056 |  | -0.193 | 0.026 |  | 0.133 |
|  | effort level5:timepoint5:depression group | -0.035 | 0.056 |  | -0.144 | 0.075 |  | 0.535 |
|  | effort level1:timepoint6:depression group | -0.039 | 0.056 |  | -0.148 | 0.070 |  | 0.482 |
|  | effort level2:timepoint6:depression group | 0.075 | 0.056 |  | -0.034 | 0.184 |  | 0.176 |
|  | effort level3:timepoint6:depression group | -0.071 | 0.056 |  | -0.180 | 0.038 |  | 0.200 |
|  | effort level4:timepoint6:depression group | -0.077 | 0.056 |  | -0.186 | 0.032 |  | 0.167 |
|  | effort level5:timepoint6:depression group | -0.058 | 0.056 |  | -0.167 | 0.051 |  | 0.299 |

**Table S3. Regression of subjective values on effort level and effort timing, group variables in Experiment 3.** Effort level 6, Timepoint 1 and Control group served as reference levels in treatment contrast coding. Effort levels corresponded to task difficulty in the N-back task, ranging from 1-back (effort level 1) to 6-back (effort level 6); Timepoints 1–6 represent increasing delays in effort exertion (timepoint 1: right now; timepoint 2: in 1 day; timepoint 3: in 3 days; timepoint 4: in 1 week; timepoint 5: in 2 weeks; timepoint 6: in 1 month). Estimates reflect the change in SV relative to the reference levels; positive estimates indicate higher SV. The model included random intercepts and random slopes for effort level by subjects as random effects. Bold p-values indicate p < .05.
